
JOURNAL INFORMATION
==============================
NLM Title Abbreviation: Orthop Surg
Journal ID: Orthop Surg
NLM Journal ID: 101501666
Journal ID: OS

Orthopaedic Surgery

ISSN: 1757-7853
EISSN: 1757-7861
Publisher: Wiley

ARTICLE INFORMATION
==============================
PMCID: PMC11474746
DOI: 10.1111/os.13343
Article ID: OS13343
Article version: 1
Subjects: Issue Information, Issue Information

Issue Information

Electronic publication date: 2023 Sep 3
Publication date: 2023 Sep
Volume: 15
Issue: 9
First page: 2193
Last page: 2194
Copyright: © 2023 The Authors. Orthopaedic Surgery published by Tianjin Hospital and John Wiley & Sons Australia, Ltd.
License: This is an open access article under the terms of the http://creativecommons.org/licenses/by/4.0/ License, which permits use, distribution and reproduction in any medium, provided the original work is properly cited.
License URL: https://creativecommons.org/licenses/by/4.0/

Figure count: 0
Table count: 0
Page count: 5

source-schema-version-number: 2.0
cover-date: September 2023
details-of-publishers-convertor: Converter:WILEY_ML3GV2_TO_JATSPMC version:6.4.9 mode:remove_FC converted:15.10.2024

==============================

Volume 15 • Issue 9 • September 2023 • ISSN 1757-7861

### Orthopaedic Surgery

Open Access

GUIDELINE

2195
CLINICAL GUIDELINES FOR THE DIAGNOSIS AND TREATMENT OF FRAGILITY FRACTURES OF THE PELVIS

REVIEW ARTICLES

2213
RESEARCH PROGRESS ON THE PATHOGENESIS OF KNEE OSTEOARTHRITIS

2225
TIBIAL TUBERCLE-TROCHLEAR GROOVE DISTANCE HAS BETTER DIAGNOSTIC RELIABILITY THAN TUBERCLE-POSTERIOR CRUCIATE LIGAMENT DISTANCE FOR PREDICTING PATELLAR INSTABILITY: A SYSTEMATIC REVIEW

2235
POSTERIOR OLECRANON FRACTURE DISLOCATIONS IN ADULTS: A SYSTEMATIC REVIEW

2244
RESEARCH PROGRESS OF NANOMATERIALS IN CHEMOTHERAPY OF OSTEOSARCOMA

CLINICAL ARTICLES

2260
A NOVEL FREE-HAND TECHNIQUE OF PEDICLE SCREW PLACEMENT IN THE LUMBAR SPINE: ACCURACY EVALUATION AND PRELIMINARY CLINICAL RESULTS

2267
EPIDEMIOLOGICAL CHARACTERISTICS AND TRENDS OF PRIMARY HIP ARTHROPLASTY IN FIVE TERTIARY HOSPITALS: A MULTICENTER RETROSPECTIVE STUDY

2274
PSOAS MAJOR SWELLING GRADE AFFECTS THE CLINICAL OUTCOMES AFTER OLIF: A RETROSPECTIVE STUDY OF 89 PATIENTS

2283
EFFICACY OF ADDING ACETAMINOPHEN TO PREEMPTIVE MULTIMODAL ANALGESIA IN TOTAL KNEE ARTHROPLASTY: A DOUBLE-BLINDED RANDOMIZED STUDY

2291
HIGH ENDPLATE HOUNSFIELD UNITS VALUE INDICATE INTERVERTEBRAL DISC DEGENERATION FOLLOWING TRANSFORAMINAL LUMBAR INTERBODY FUSION SURGERY

2300
TREATMENT OF ANTERIOR DISLOCATION OF THE SACROILIAC JOINT *VIA* THE LATERAL-RECTUS APPROACH: SURGICAL TECHNIQUES AND PRELIMINARY OUTCOMES

2309
DOES PREOPERATIVE MODIC CHANGES INFLUENCE THE SHORT-TERM FUSION RATE OF SINGLE LEVEL TRANSFORAMINAL LUMBAR INTERBODY FUSION?—A MATCHED-PAIR CASE CONTROL STUDY

2318
LEARNING CURVE AND CLINICAL OUTCOMES OF ULTRASONIC OSTEOTOME-BASED EN BLOC LAMINECTOMY FOR THORACIC OSSIFICATION OF THE LIGAMENTUM FLAVUM

2328
AN EXCITING PERFORMANCE OF ESTABLISHED AND NOVEL BIOMARKERS IN DIAGNOSING PERIPROSTHETIC JOINT INFECTIONS: A SINGLE-CENTER RETROSPECTIVE COHORT STUDY

2334
COMPARISON OF LUMBOSACRAL FUSION GRADE IN PATIENTS AFTER TRANSFORAMINAL AND ANTERIOR LUMBAR INTERBODY FUSION WITH MINIMUM 2-YEAR FOLLOW-UP

2342
SEVERE SYMPTOMATIC EPIDURAL HEMATOMA FOLLOWING PERCUTANEOUS ENDOSCOPIC UNILATERAL LAMINECTOMY FOR BILATERAL DECOMPRESSION (ENDO-ULBD)—SERIES REPORT AND MANAGEMENT STRATEGIES

2354
DOES IMMEDIATE POSTOPERATIVE EARLY AMBULATION AFFECT CLINICAL RESULTS OF FULL-ENDOSCOPIC LUMBAR DISCECTOMY? A HISTORICAL CONTROL STUDY OF DAYTIME OPERATION WITH A 8-HOUR HOSPITAL STAY VERSUS INPATIENT OPERATION

2363
A COMPARISON OF OUTCOMES BETWEEN THE WILTSE APPROACH WITH PEDICLE SCREW FIXATION AND THE PERCUTANEOUS PEDICLE SCREW FIXATION FOR MULTI-SEGMENTAL THORACOLUMBAR FRACTURES

2373
THE ROLE OF GLOBAL FEMORAL OFFSET IN TOTAL HIP ARTHROPLASTY WITH HIGH HIP CENTER TECHNIQUE

2383
THREE-DIMENSIONAL PRINTED PLATE TEMPLATE VERSUS HEMIPELVIS MODEL IN PATIENT-SPECIFIC PLATE PREPARATION FOR POSTERIOR WALL ACETABULAR FRACTURES

2393
ARTHROSCOPY CONFERS FAVORABLE CLINICAL OUTCOMES IN ASIAN PATIENTS WITH BORDERLINE DEVELOPMENTAL DYSPLASIA OF THE HIP

2400
BOTH-COLUMN ACETABULAR FRACTURES WITH POSTERIOR WALL INVOLVED CAN BE MANAGED THROUGH SINGLE ANTERIOR APPROACH BY EVALUATION OF COMPUTER-ASSISTED VIRTUAL SURGERY TECHNIQUE

RESEARCH ARTICLES

2410
ANGIOPOIETIN-2 PROMOTES MECHANICAL STRESS-INDUCED EXTRACELLULAR MATRIX DEGRADATION IN ANNULUS FIBROSUS *VIA* THE HIF-1α/NF-κB SIGNALING PATHWAY

2423
ASPN SYNERGIZES WITH HAPLN1 TO INHIBIT THE OSTEOGENIC DIFFERENTIATION OF BONE MARROW MESENCHYMAL STROMAL CELLS AND EXTRACELLULAR MATRIX MINERALIZATION OF OSTEOBLASTS

2435
IPSILATERAL FIXATION AND RECONSTRUCTION OF THE CERVICAL SPINE AFTER RESECTION OF A DUMBBELL TUMOR VIA A UNILATERAL POSTERIOR APPROACH: A CASE REPORT AND BIOMECHANICAL STUDY

OPERATIVE TECHNIQUE

2445
HOW TO PERFORM BETTER ON OXFORD UKA? A TECHNICAL NOTE FROM OVER 500 SURGICAL EXPERIENCES

BRIEF REPORT

2454
DOUBLE VERTEBRAE KÜMMELL DISEASE: FIVE CASES REPORT AND LITERATURE REVIEW

CASE REPORTS

2464
COMPUTER-ASSISTED NAVIGATION FULL VISUALIZATION SPINAL ENDOSCOPIC SURGERY FOR LUMBAR VERTEBRAL OSTEOID OSTEOMA

2471
INTRA-ARTICULAR OSTEOID OSTEOMA OF THE INTERMEDIATE CUNEIFORM BONE CAUSING ARTICULAR DEGENERATION: A RARE CASE AND LITERATURE REVIEW

2477
COEXISTING KIENBÖCK'S AND PREISER'S DISEASE OF THE WRIST: EXPERIENCE WITH PROXIMAL ROW CARPECTOMY WITH DORSAL CAPSULAR INTERPOSITION TECHNIQUE

WILEY

View this journal online at wileyonlinelibrary.com/journal/os

### Orthopaedic Surgery

**Editor-in-Chief**
Gui-xing Qiu, *Beijing, China*
Francis John Hornicek, *Boston, USA*

**Associate Editor**
Yong-cheng Hu, *Tianjin, China*
Thomas Blattert, *Leipzig, Germany*
Douglas Letson, *Tampa, USA*
Xin-long Ma, *Tianjin, China*
Ling Qin, *Hong Kong, China*

**Editorial Board**
Giovanni Alessi, *Gent, Belgium*
Haluk Berk, *Izmir, Turkey*
Thomas Blattert, *Leipzig, Germany*
Mathis Bostrom, *New York, USA*
Kao-wha Chang, *Taiwan, China*
Edmund YS Chao, *Baltimore, USA*
Liao-bin Chen, *Wuhan, China*
Yan-xi Chen, *Shanghai, China*
Kenneth MC Cheung, *Hong Kong, China*
Thomas L. Clemens, *Birmingham, USA*
Benny Thellefsen Dahl, *Copenhagen, Denmark*
Lian-fu Deng, *Shanghai, China*
Jean Destandau, *Bordeaux, France*
Jian Dong, *Shanghai, China*
Shun-wu Fan, *Hangzhou, China*
David Fang, *Hong Kong, China*
Shi-jun Gao, *Shijiazhuang, China*
Wei Guo, *Beijing, China*
Yong Hai, *Beijing, China*
Alan R. Hargens, *San Diego, USA*
Francis John Hornicek, *Boston, USA*
William C. Horton, *Atlanta, USA*
John Hsiang, *Seattle, USA*
Yong-cheng Hu, *Tianjin, China*
Qing Jiang, *Nanjing, China*
Paul Jutte, *Groningen, Netherlands*
Jean Pierre Kalala, *Gent, Belgium*
Choon-Ki Lee, *Seoul, Korea*
Jean Jacque Lehuec, *Bordeaux, France*
Lawrence G. Lenke, *Seattle, USA*
Douglas Letson, *Tampa, USA*
Kwok Sui Leung, *Hong Kong, China*
Jian-hao Lin, *Beijing, China*
Bao-ge Liu, *Beijing, China*
Wei-hee Lo, *Taiwan, China*
William Weijia Lu, *Hong Kong, China*
Keith DK Luk, *Hong Kong, China*
Zong-ping Luo, *Suzhou, China*
Cyril Mauffrey, *Denver, USA*
Wei-hu Ma, *Ningbo, China*
Xin-long Ma, *Tianjin, China*
Ling Qin, *Hong Kong, China*
Gui-xing Qiu, *Beijing, China*
Axel Reinhardt, *Potsdam, Germany*
Pu-yi Sheng, *Guangzhou, China*
Harry L Shufflebarger, *Miami, USA*
David Stulberg, *Chicago, USA*
Paul Sung, *San Diego, USA*
Paul Unwin, *Leicester, UK*
Luc F. De Waele, *Gent, Belgium*
Fei Wang, *Shijiazhuang, China*
Wei-jun Wang, *Nanjing, China*
James N. Weinstein, *Chicago, USA*
Hee-Kit Wong, *Singapore*
Su-jia Wu, *Nanjing, China*
Jian-ru Xiao, *Shanghai, China*
Hui-lin Yang, *Suzhou, China*
Tzai-chiu Yu, *Taiwan, China*
Xi-jie Yu, *Chengdu, China*
Hansen A. Yuan, *New York, USA*
Hong-wen Zhu, *New York, USA*

**Address for Editorial Correspondence:** Editorial Board of *Orthopaedic Surgery*, Tianjin Hospital, 122 Munan Road, Tianjin 300050, China (email: orthopaedicsurgery@126.com; telephone: +86 22 2833 4734; fax: +86 22 2824 1184).

**Disclaimer:** The Publisher, Tianjin Hospital and Editors cannot be held responsible for errors or any consequences arising from the use of information contained in this journal; the views and opinions expressed do not necessarily reflect those of the Publisher, Tianjin Hospital and Editors, neither does the publication of advertisements constitute any endorsement by the Publisher, Tianjin Hospital and Editors of the products advertised.

**Copyright © 2023 The Authors. Orthopaedic Surgery published by Tianjin Hospital and John Wiley & Sons Australia, Ltd**

**Copyright and Copying (in any format)**
Copyright © 2023 The Authors. Orthopaedic Surgery published by Tianjin Hospital and John Wiley & Sons Australia, Ltd. All rights reserved. No part of this publication may be reproduced, stored or transmitted in any form or by any means without the prior permission in writing from the copyright holder. Authorization to copy items for internal and personal use is granted by the copyright holder for libraries and other users registered with their local Reproduction Rights Organisation (RRO), e.g. Copyright Clearance Center (CCC), 222 Rosewood Drive, Danvers, MA 01923, USA (www.copyright.com), provided the appropriate fee is paid directly to the RRO. This consent does not extend to other kinds of copying such as copying for general distribution, for advertising or promotional purposes, for republication, for creating new collective works or for resale. Permissions for such reuse can be obtained using the RightsLink “Request Permissions” link on Wiley Online Library. Special requests should be addressed to: permissions@wiley.com

For submission instructions, subscription and all other information visit: http://wileyonlinelibrary.com/journal/os

ISSN 1757-7861 (Online)

OS.JEB.September23
